# Obituary

**Published:** 1841-07

**Authors:** 


### OBITUARY.

If we but seldom mention the death of Medical gentlemen, it is from no disrespect to their memories, but through inadvertence, or ignorance of the event.

Died, at Marietta, O. on the 27th of May, of Pulmonary Consumption, John C. Stone, M. D. a native of that State, and a young physician of respectable promise.

